# Identifying potential pharmacological targets and molecular pathways of Meliae cortex for COVID-19 therapy

**DOI:** 10.3389/fimmu.2023.1128164

**Published:** 2023-02-02

**Authors:** Shakeel Ahmad Khan, Terence Kin Wah Lee

**Affiliations:** ^1^ Department of Applied Biology and Chemical Technology, The Hong Kong Polytechnic University, Kowloon, Hong Kong SAR, China; ^2^ Research Center for Chinese Medicine Innovation, The Hong Kong Polytechnic University, Kowloon, Hong Kong SAR, China; ^3^ State Key Laboratory of Chemical Biology and Drug Discovery, The Hong Kong Polytechnic University, Kowloon, Hong Kong SAR, China

**Keywords:** Meliae cortex, phytonutrients, pharmacology, docking, targets, COVID-19

## Abstract

Coronavirus disease-19 (COVID-19), caused by SARS-CoV-2, has contributed to a significant increase in mortality. Proinflammatory cytokine-mediated cytokine release syndrome (CRS) contributes significantly to COVID-19. Meliae cortex has been reported for its several ethnomedical applications in the Chinese Pharmacopoeia. In combination with other traditional Chinese medicines (TCM), the Meliae cortex suppresses coronavirus. Due to its phytoconstituents and anti-inflammatory capabilities, we postulated that the Meliae cortex could be a potential therapeutic for treating COVID-19. The active phytonutrients, molecular targets, and pathways of the Meliae cortex have not been explored yet for COVID-19 therapy. We performed network pharmacology analysis to determine the active phytoconstituents, molecular targets, and pathways of the Meliae cortex for COVID-19 treatment. 15 active phytonutrients of the Meliae cortex and 451 their potential gene targets were retrieved from the Traditional Chinese Medicine Systems Pharmacology (TCMSP) and SwissTargetPrediction website tool, respectively. 1745 COVID-19-related gene targets were recovered from the GeneCards. 104 intersection gene targets were determined by performing VENNY analysis. Using the DAVID tool, gene ontology (GO) and KEGG pathway enrichment analysis were performed on the intersection gene targets. Using the Cytoscape software, the PPI and MCODE analyses were carried out on the intersection gene targets, which resulted in 41 potential anti-COVID-19 core targets. Molecular docking was performed with AutoDock Vina. The 10 anti-COVID-19 core targets (AKT1, TNF, HSP90AA1, IL-6, mTOR, EGFR, CASP3, HIF1A, MAPK3, and MAPK1), three molecular pathways (the PI3K-Akt signaling pathway, the HIF-1 signaling pathway, and the pathways in cancer) and three active phytonutrients (4,8-dimethoxy-1-vinyl-beta-carboline, Trichilinin D, and Nimbolin B) were identified as molecular targets, molecular pathways, and key active phytonutrients of the Meliae cortex, respectively that significantly contribute to alleviating COVID-19. Molecular docking analysis further corroborated that three Meliae cortex’s key active phytonutrients may ameliorate COVID-19 disease by modulating identified targets. Hence, this research offers a solid theoretic foundation for the future development of anti-COVID-19 therapeutics based on the phytonutrients of the Meliae cortex.

## 1 Introduction

**Figure 1:**
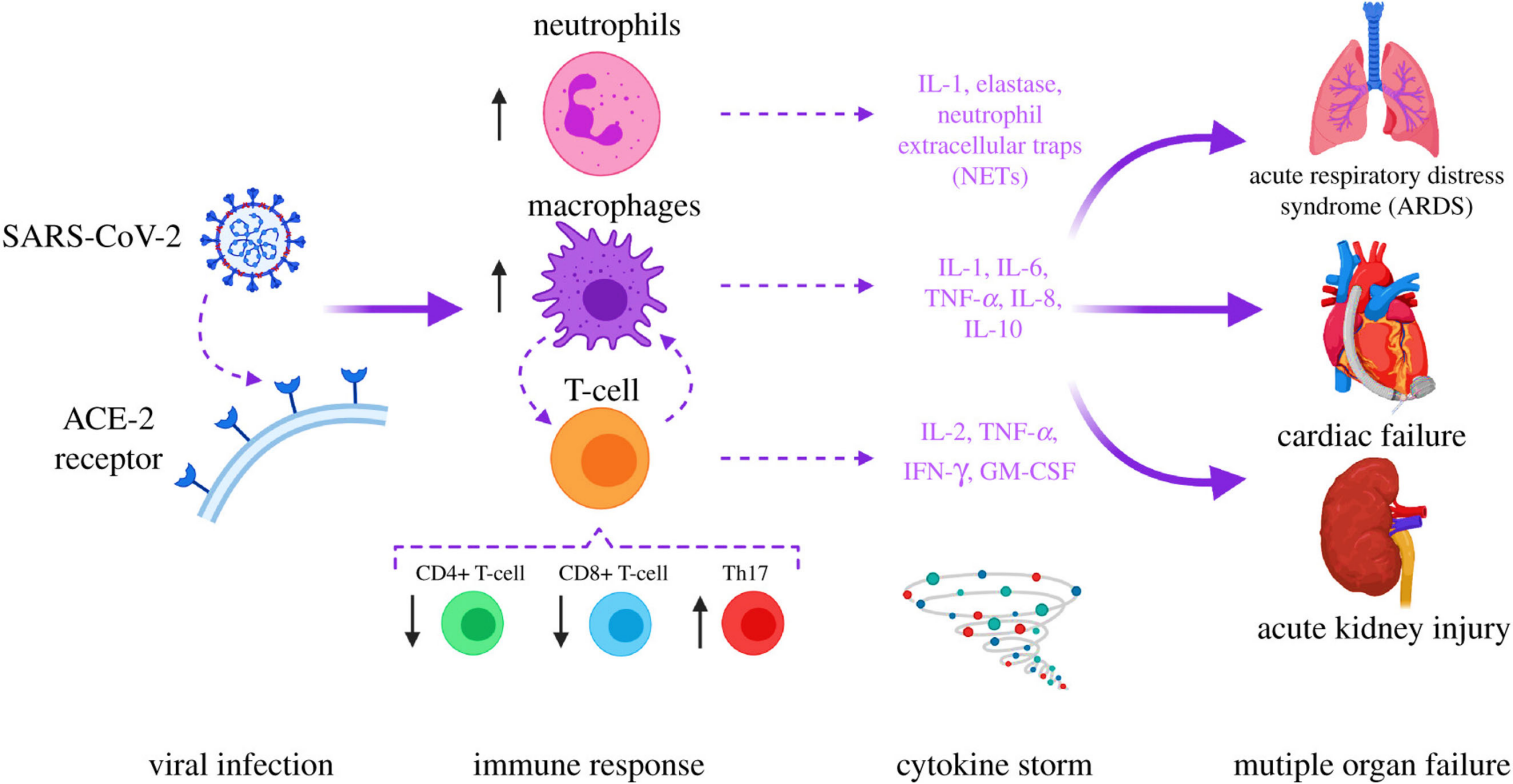
SARS-COV-2 in the etiology of COVID-19. Acute respiratory distress syndrome (ARDS) and multiple organ dysfunction syndromes (MODS) are mediated by cytokines release syndrome (CRS). The image was reproduced from (5) under CC BY 4.0.

The worldwide spread of the lethal SARS-CoV-2 that induces coronavirus disease-19 (COVID-19) has contributed to a significant increase in mortality (1). As of January 22, 2023, the number of patients had reached 673,282,002, and at least 6,745,967 fatalities have occurred (2). A high death rate in COVID-19 patients has been reported due to multiple organ dysfunction syndromes (MODS) and acute respiratory distress syndrome (ARDS) (3, 4) (**Figure 1**). Numerous reports have shown that cytokine release syndrome (CRS) contributes to ARDS and MODS, which is recognized as the prime inducer of SARS-COV and MERS-CoV contagion in humans (6, 7). CRS in COVID-19 patients has been reported to induce many pro-inflammatory mediators, including TNF, monocyte chemoattractant protein-1a (MCP-1), inducible protein 10 (IP-10), IFN-α, GM-CSF, interleukin (IL)-1, IL-2, IL-6, and IL-7 (8–12). IL-6 correlates with viral load and is highest before mechanical ventilation (5, 13). Tocilizumab, an IL-6 receptor antagonist, has been shown in studies to be useful in reducing cytokine storms in COVID-19 patients. Nevertheless, preliminary data from randomized clinical trials are still not definitive (14–16). Reports also indicate adverse effects (drug-induced liver damage and rise in liver enzymes) in COVID-19 patients after the administration of tocilizumab (17, 18). In addition, certain therapeutic combinations (Baricitinib with remdesivir and tofacitinib with glucocorticoids) have shown modest benefits in lowering recovery time and respiratory failure, respectively (12, 19, 20). Therefore, in this instance, developing potential therapeutic anti-inflammatory medications targeting pro-inflammatory mediators with minimized adverse events for the treatment of COVID-19 remains a pressing need.

Effective measures, such as bioactive natural products (21, 22) and small-molecule inhibitors (23–25), are greatly needed against the Omicron variant. However, promising magic bullets still do not exist (26, 27). As an indispensable resource, traditional Chinese medicines (TCM) have demonstrated potential value in countering COVID-19 (28). Furthermore, in comparison to conventional therapeutic medications, TCM’s capacity to alleviate just the symptoms of an illness without harming healthy cells offers it an increasingly desirable option for the development of new drugs (6, 29, 30).

Meliae cortex is the dried bark of Melia azedarach Linn., also known in Korea and China as Go-Ryun-Pi and Kulianpi, respectively (31, 32). In the Chinese Pharmacopoeia, the Meliae cortex has been reported to be used as an insect repellant and as a therapeutic for treating Tinea imbricata (33). Meliae cortex has been used for several ethnomedical applications, such as antidiarrheal, deobstruent, diuretic, rheumatic pain, stomachache, fever aches, and pains, etc. (34). Pharmacological investigations show that extracts and components of the Meliae cortex also have anti-diabetic, anti-allergic, anti-inflammatory, anticancer, antioxidant, and antibacterial effects (31, 33, 35, 36). In conjunction with other TCM, the Meliae cortex has been found to alleviate acute pancreatitis and suppress the replication of coronavirus and mouse hepatitis virus A59 (MHV-A59) (37–39). Despite these findings against viruses, there is currently a dearth of evidence suggesting that the Meliae cortex might play a crucial role in COVID-19 management. Therefore, based on the existence of phytoconstituents and their anti-inflammatory properties, we hypothesized that the Meliae cortex could be a potential therapeutic for treating COVID-19. Moreover, the active phytonutrients, molecular targets, and pathways of the Meliae cortex have not been explored yet for treating COVID-19.

**Figure 2:**
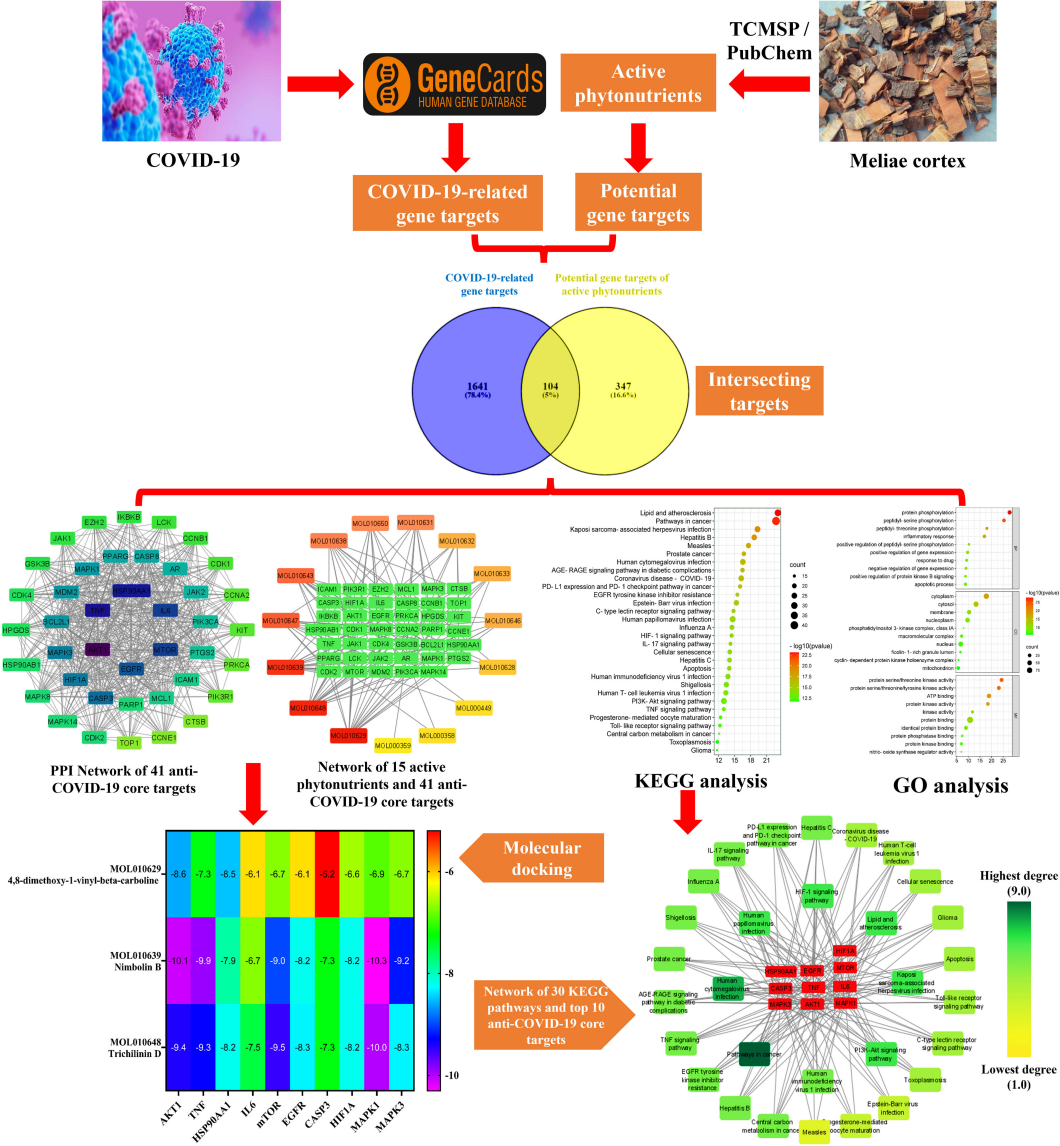
Workflow of the current investigation to identify active phytonutrients, molecular targets, and pathways of Meliae cortex for COVID-19 therapy.

Network pharmacology emerged quickly as a prominent TCM research technique based on the multidisciplinary holistic study of biological systems. Network pharmacology employs artificial intelligence and big data to determine active pharmacological components and comprehend the action mechanism of TCM (40). In the present research, we applied network pharmacology and molecular docking techniques to investigate the pharmacologically active phytonutrients, molecular targets, and action mechanisms of the Meliae cortex implicated in treating COVID-19, and the workflow of this study is presented in **Figure 2**.

## 2 Materials and methods

### 2.1 Software and databases

Different open-source software and databases including TCMSP (Version 2.3, https://old.tcmsp-e.com/tcmsp.php, accessed on June 10, 2022), GeneCards^®^: The Human Gene Database (https://www.genecards.org/, accessed on June 10, 2022), Venny 2.1 (https://bioinfogp.cnb.csic.es/tools/venny/, accessed on June 10, 2022), SwissTargetPrediction (http://www.swisstargetprediction.ch/, accessed on June 10, 2022), STRING (https://string-db.org/, version 11.5, accessed on June 10, 2022), Cytoscape (version 3.9.0, Boston, MA, USA, accessed on June 10, 2022), Cystoscope’s Molecular Complex Detection (MCODE) plug-in (accessed on June 10, 2022), DAVID (https://david.ncifcrf.gov/, Version 6.8, accessed on June 10, 2022), Bioinformatics platform (http://www.bioinformatics.com.cn/, accessed on June 10, 2022), Protein Data Bank (https://www.rcsb.org/, accessed on June 14, 2022), NCBI PubChem (https://pubchem.ncbi.nlm.nih.gov/, accessed on June 14, 2022), ChemDraw ultra-12.0 (accessed on June 16, 2022), BIOVIA Discovery Studio Visualizer 2021 (accessed on June 16, 2022), AutoDock Vina (Version 1.2.0, accessed on June 16, 2022), were employed in this research work.

### 2.2 Collection of Meliae cortex’s active phytonutrients

The phytonutrients of the Meliae cortex were retrieved by utilizing the TCMSP (41). TCMSP was developed using the systems pharmacology framework for herbal medicines by correlating each component’s ADME characteristics. The retrieved phytonutrients of the Meliae cortex that fulfilled both the drug-likeness (DL) of at least 0.18 and the oral bioavailability (OB) of at least 30% were selected for further investigation, and they are named active phytonutrients.

### 2.3 Identification of COVID-19-related gene targets

The COVID-19-related gene targets were identified by searching the keywords “Novel Coronavirus” and “Novel Coronavirus Pneumonia” in the GeneCards^®^: The Human Gene Database (42). By employing Venny 2.1 platform (43), duplicate gene targets were eliminated after merging the gene targets of both keywords.

### 2.4 Prediction of potential gene targets of Meliae cortex’s active phytonutrients

The potential gene targets of the Meliae cortex’s active phytonutrients were determined using the SwissTargetPrediction database (44). Potential gene targets with probabilities greater than zero were chosen for further investigation.

### 2.5 Determination of intersection gene targets

The Venny 2.1 online database (43) was used to identify the intersection gene targets between the potential gene targets of the Meliae cortex’s active phytonutrients and the COVID-19-related gene targets. Intersecting gene targets were considered potential anti-COVID-19 prime targets.

### 2.6 Protein-protein interaction analysis

The potential anti-COVID-19 prime targets were then uploaded to the STRING database for PPI analysis while maintaining a confidence score of > 0.4 and a species threshold of “Homo sapiens” (45, 46). The PPI analysis findings from STRING were then uploaded in tab-separated values (tsv.) file format into Cytoscape software for exploring the potential anti-COVID-19 core targets (47).

### 2.7 Molecular complex detection analysis

The critical modules in the PPI network of potential anti-COVID-19 prime targets were then identified using Cystoscope’s MCODE) plug-in (47). Conditions for MCODE analysis were as follows: Find clusters: in the whole network, Degree cutoff: 2, Node score cutoff: 0.2, K-core: 2, and Maximum Depth: 100 (48).

### 2.8 Network construction between the Meliae cortex’s active phytonutrients and the potential COVID-19-related (prime and core) targets

Using Cytoscape software, the network between the active phytonutrients of the Meliae cortex and the COVID-19-related (prime and core) targets was established (46, 47).

### 2.9 GO and KEGG enrichment analysis

Using the DAVID database, the GO functional and KEGG pathway enrichment analyses were further performed on potential anti-COVID-19 prime targets by keeping the species threshold of “Homo sapiens” (49, 50). There are three categories of GO terms: cellular component (CC), biological process (BP), and molecular function (MF). By uploading the data to a Bioinformatics platform, the top 10 GO analysis data (BP, CC, and MF) and the top 30 KEGG pathways were shown in the form of an enrichment dot bubble (51). Utilizing the traditional hypergeometric test, statistical significance was determined. After controlling the false discovery rate (FDR) for multiple hypothesis testing using the Benjamini–Hochberg technique, the adjusted *p* < 0.05 was employed as the significant level in our analysis (46).

### 2.10 Molecular docking

The current study relates the functioning of the Meliae cortex’s active phytonutrients, and the potential anti-COVID-19 core targets. The top three Meliae cortex’s active phytonutrients were assessed for their binding affinity with the top ten potential anti-COVID-19 core targets (AKT1, TNF, HSP90AA1, IL-6, mTOR, EGFR, CASP3, HIF1A, MAPK3, and MAPK1). The crystal structures of all the core targets (PDB IDs: 3O96, 5MU8, 4BQG, 1ALU, 4JSV, 5Y9T, 1NME, 1H2M, 6GES, and 6G9K), respectively, were retrieved in PDB format from the Protein Data Bank (52). The chemical structures of three of the Meliae cortex’s active phytonutrients (MOL010629, MOL010648, and MOL010639) were sketched in ChemDraw and verified from TCMPS and NCBI PubChem (41, 53). After, their two-dimensional (2D) structures were saved in Mol2 file format and uploaded to BIOVIA Discovery Studio Visualizer, and constructed their three-dimensional (3D) structures in PDB format (54). The crystal structures of all the core targets in PDB format are then uploaded to BIOVIA Discovery Studio Visualizer and extracted the heteroatoms (water and other ligands) and subsequently added the polar hydrogens. Finally, the resultant proteins were saved in PDB format, imported to AutoDock Vina, and added the Kollman and Gasteiger partial charges (55). The formatted 3D structures of the Meliae cortex’s active phytonutrients in PDB were then imported to AutoDock Vina, checked for torsions, and saved in pdbqt format. The uploaded phytonutrients and proteins are selected as ligands and macromolecules, respectively, and later saved in pdbqt format. A grid box was then construed for each protein for blind docking using AutoDock Vina. The scripts were then written for molecular docking using a command prompt, and acquired results were presented in the form of binding affinity. The docked complexes were further visualized with BIOVIA Discovery Studio Visualizer, and 2D and 3D images were generated.

## 3 Results

### 3.1 Screening of Meliae cortex’s active phytonutrients

The TCMSP database yielded a total of 114 phytonutrients from the Meliae cortex. Additionally, based on OB of at least 30% and DL of at least 0.18, active phytonutrient screening was performed, and the Meliae cortex contained 15 active phytonutrients, as shown in **Table 1**.

**Table 1:** The list of Meliae cortex’s active phytonutrients.

IDs	Phytonutrients Name	Structures	OB	DL
MOL010628	(+)-Syringaresinol-di-O-β-D-glucosid _qt	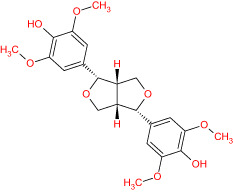	34.99	0.72
MOL010629	4,8-dimethoxy-1-vinyl-beta-carboline	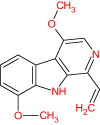	66.78	0.20
MOL010631	Kssulactone	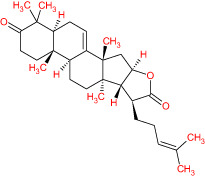	45.44	0.81
MOL010632	Kulinone	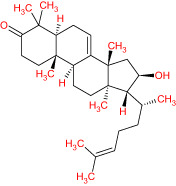	44.88	0.77
MOL010633	Kulolactone	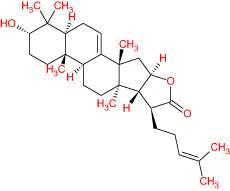	43.97	0.81
MOL010638	Nimbolin A	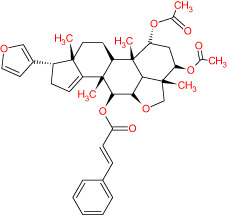	32.11	0.34
MOL010639	Nimbolin B	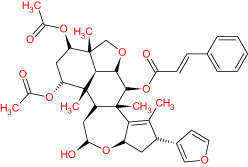	30.54	0.30
MOL010643	Sendanolactone	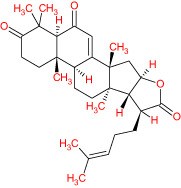	63.18	0.90
MOL010646	Trichilin A	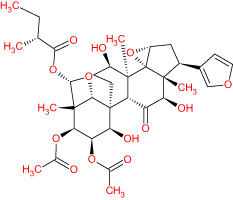	39.59	0.28
MOL010647	Trichilinin B	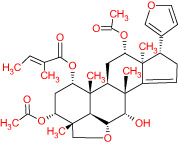	30.74	0.47
MOL010648	Trichilinin D	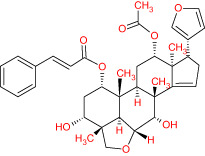	33.51	0.34
MOL010650	SMR000232316	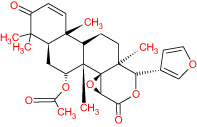	35.43	0.74
MOL000358	Beta-sitosterol	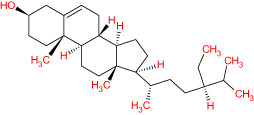	36.91	0.75
MOL000359	Sitosterol	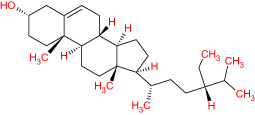	36.91	0.75
MOL000449	Stigmasterol	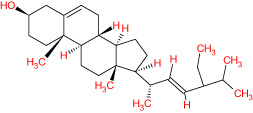	43.83	0.76

### 3.2 Potential gene targets of Meliae cortex’s active phytonutrients

A SwissTargetPrediction online database was used to identify potential gene targets of Meliae cortex’s active phytonutrients. With a probability value greater than 0, 1044 potential gene targets for 15 active phytonutrients were chosen. After deleting duplication, 451 potential gene targets were chosen for further investigation.

### 3.3 COVID-19-related gene targets

A total of 1745 COVID-19-related gene targets were recuperated from the GeneCards database by searching the keywords “Novel Coronavirus” and “Novel Coronavirus Pneumonia.”

### 3.4 Intersection gene targets analysis

**Figure 3:**
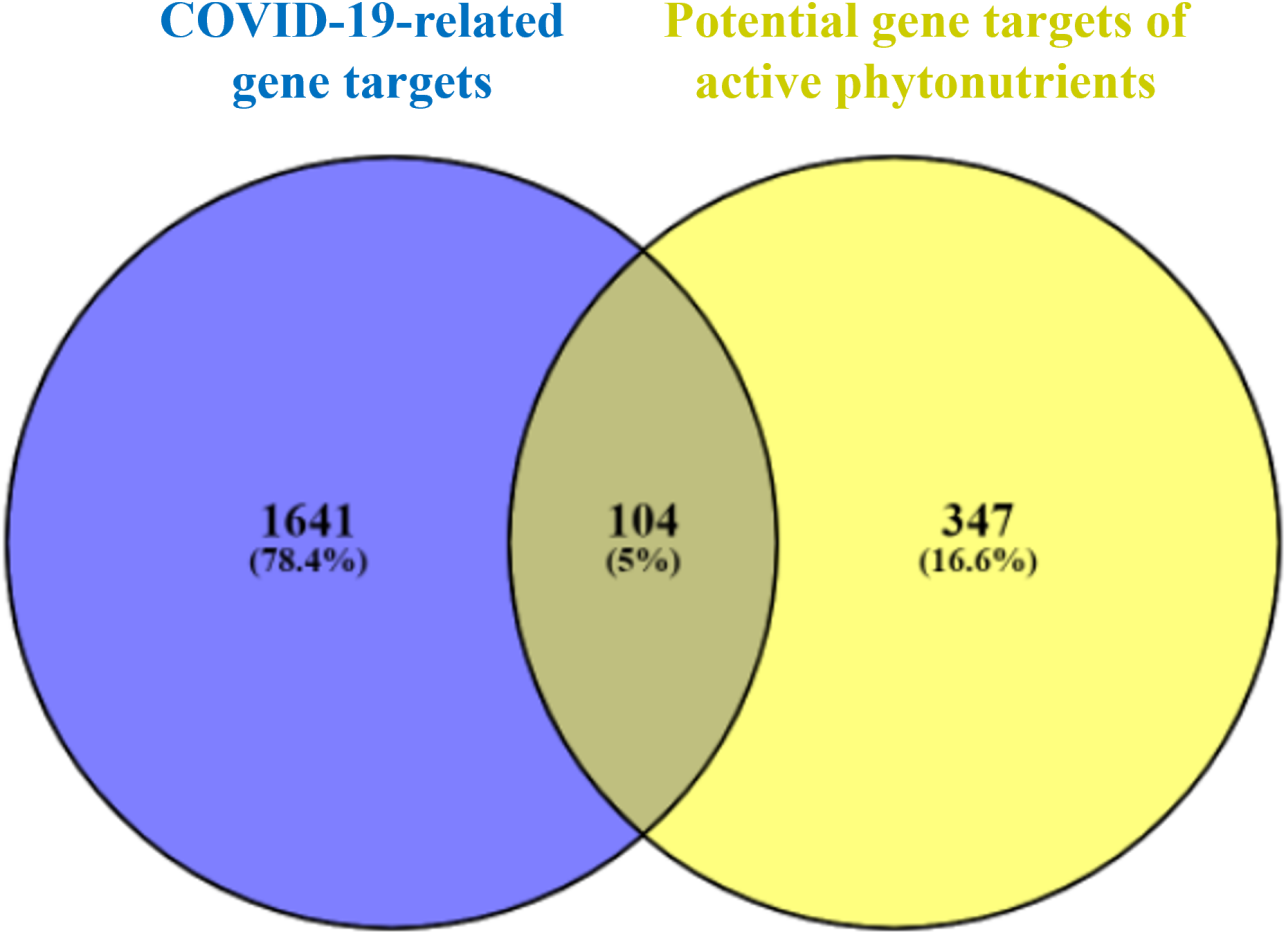
Venn diagram illustrating the relationship between the COVID-19-related gene targets and potential gene targets of Meliae cortex’s active phytonutrients, as well as their intersecting gene targets.

As shown in **Figure 3**, 104 intersecting gene targets were identified among the 451 potential gene targets of Meliae cortex’s active phytonutrients and the 1745 COVID-19-related gene targets. They were regarded as potential anti-COVID-19 prime targets.

### 3.5 PPI network analysis

**Figure 4:**
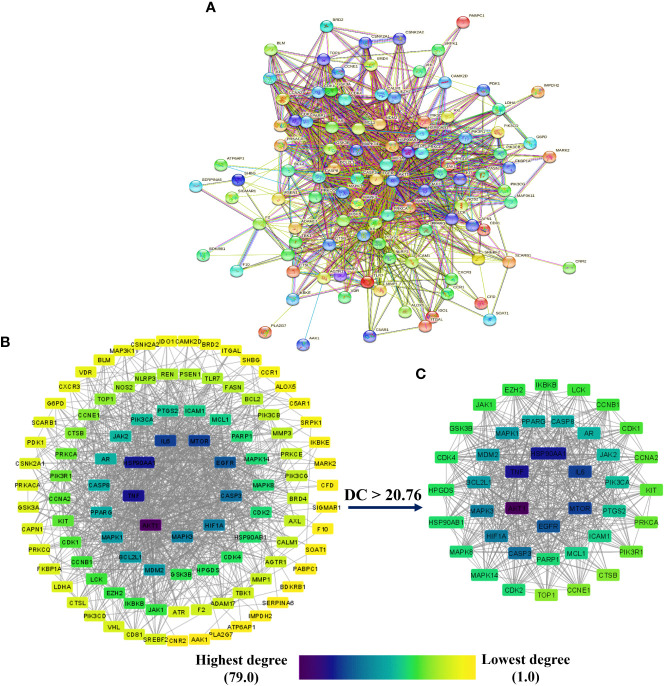
**(A)** STRING PPI interaction network. PPI interaction network of **(B)** 104 potential anti-COVID-19 prime targets and **(C)** 41 potential anti-COVID-19 core targets. The hue shifts from yellow to purple as the degree of each node increases. DC = degree centrality.

**Figure 5:**
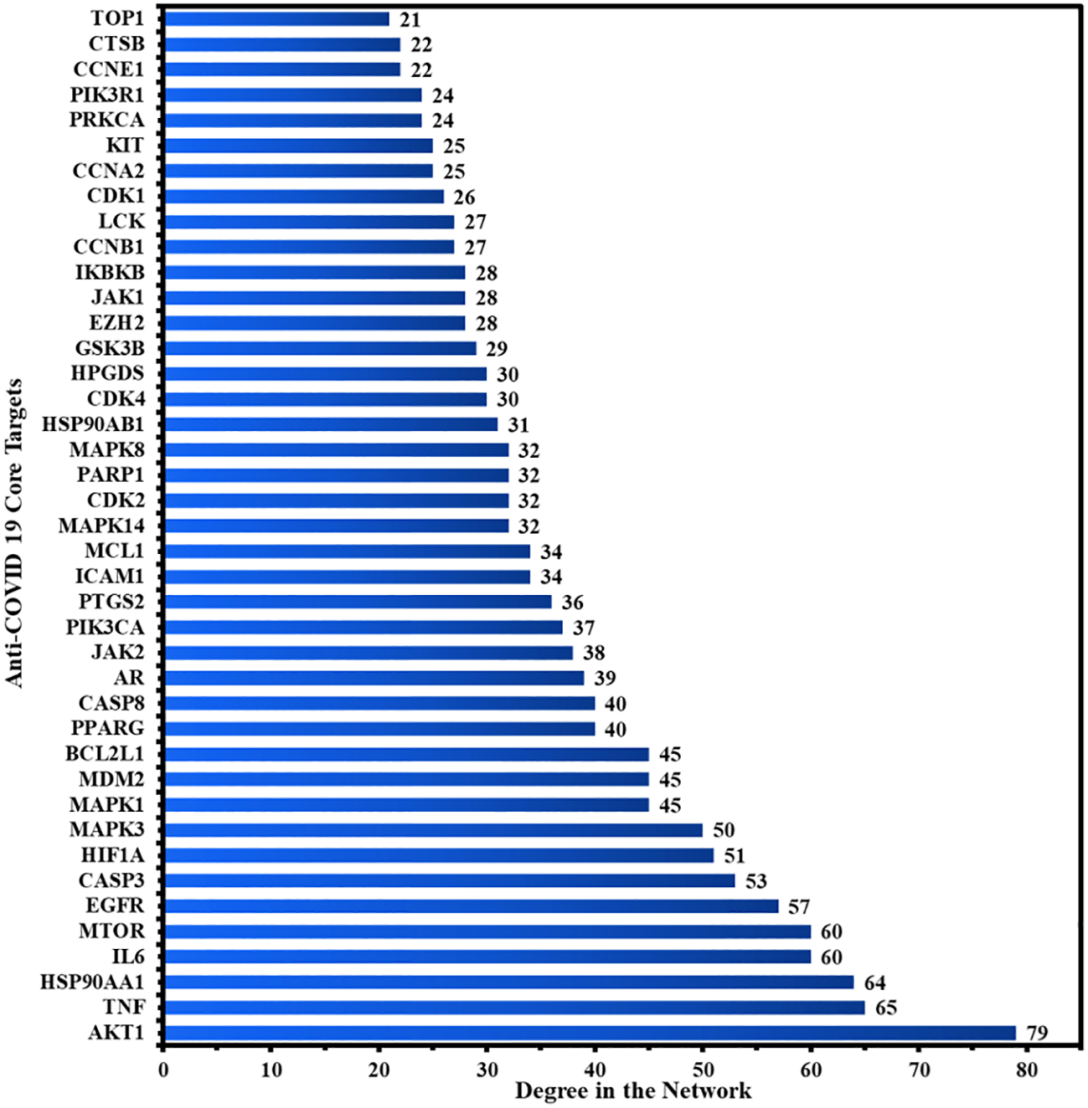
The 41 potential anti-COVID-19 core targets with their degree values.

**Figure 4A** depicts the STRING analysis result, which demonstrates that the PPI network comprises 104 nodes and 1080 edges. The average degree of the node, the average local clustering coefficient, the expected number of edges, and the average PPI enrichment p-values were 20.8, 0.66, 418, and *p* < 0.00001, respectively. As shown in **Figure 4B**, the Cytoscape analysis results demonstrated that the PPI network had 104 nodes and 1080 edges, with a characteristics path length of 1.946 between all node pairs. Furthermore, the density, diameter, average number of neighbors, clustering coefficient, network heterogeneity, network centralization, and network radius were 0.202, 4, 20.769, 0.632, 0.803, 0.576, and 2, respectively. Each node’s color changes from yellow to purple as the degree increases. The 41 nodes that comply with the criterion of degree centrality (DC) ≥ average value (20.76) were further extracted and designated as potential anti-COVID-19 core targets (**Figure 4C**). The 41 potential anti-COVID-19 core targets ranked by DC are shown in **Figure 5** as a bar graph. The top ten anti-COVID-19 core targets, AKT1, TNF, HSP90AA1, IL-6, mTOR, EGFR, CASP3, HIF1A, MAPK3, and MAPK1, were chosen for molecular docking studies with the Meliae cortex’s key active phytonutrients determined in section 3.7.

### 3.6 Cluster network analysis

**Figure 6:**
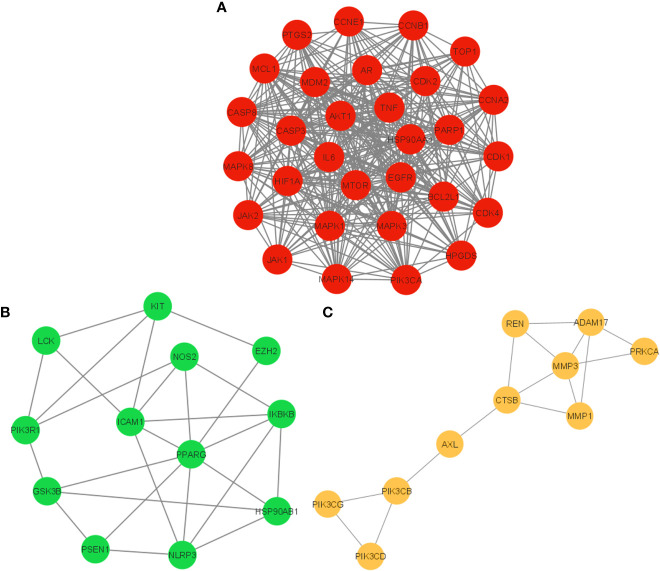
Cluster network analysis. **(A)** Cluster network 1, **(B)** Cluster network 2, and **(C)** Cluster network 3 generated using the MCODE plug-in for Cytoscape.

The PPI network of 104 potential anti-COVID-19 prime targets was further assessed for their cluster network analysis using the MCODE plug-in for Cytoscape. **Figure 6** illustrates that the PPI network of anti-COVID-19 prime targets generated three cluster networks. Cluster network 1 has 30 nodes and 356 edges, scoring 24.552 (**Figure 6A**). Cluster network 2 consists of 12 nodes and 26 edges and has a score of 4.7277 (**Figure 6B**). Cluster network 3 contains ten nodes and fifteen edges with a score of 3.333 (**Figure 6C**). In cluster network 1, BCL2L1, CASP3, MDM2, AKT1, HIF1A, mTOR, HSP90AA1, EGFR, CASP8, MAPK3, AR, MAPK1, MCL1, and CDK2 are highly interconnected with multiple gene targets and have a DC ≥ average value of (23.733). In cluster network 2, PPARG, ICAM1, NLRP3, and IKBKB are interconnected with multiple gene targets and have a DC ≥ average value of (4.333). In cluster network 3, MMP3, ADAM17, CTSB, REN, MMP1, and PIK3CB are interconnected with multiple gene targets and have a DC ≥ average value of (3.0). In addition, cluster 1 confirms the existence of the top ten potential anti-COVID-19 core targets (AKT1, TNF, HSP90AA1, IL-6, mTOR, EGFR, CASP3, HIF1A, MAPK3, and MAPK1); consequently, the results of the PPI and cluster network analyses are congruent.

### 3.7 Network of Meliae cortex’s active phytonutrients and anti-COVID-19 targets

**Figure 7:**
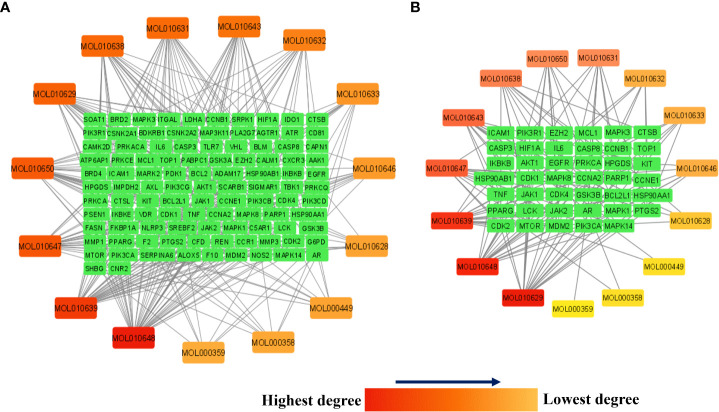
**(A)** Network of Meliae cortex’s active phytonutrients and 104 potential anti-COVID-19 prime targets. **(B)** The hub network of Meliae cortex’s active phytonutrients and 41 potential anti-COVID-19 core targets. The hue shifts from yellow to red as the node’s degree increases.

**Figure 7A** presents the network between the Meliae cortex’s active phytonutrients, and 104 potential anti-COVID-19 prime targets. The network has 119 nodes and 295 edges. The network’s diameter, radius, density, heterogeneity, and centralization were six, three, 0.037, 1.458, and 0.301, respectively. Furthermore, the average number of neighbors, characteristic path length, clustering coefficient, and connected components were 4.630, 3.190, 0.000, and 1, respectively. Each edge denotes the interaction between the Meliae cortex’s active phytonutrients and potential anti-COVID-19 prime targets. The degree of a node indicates the number of edges connecting it to other network nodes. The hue shifts from yellow to red as the node’s degree increases.

**Figure 7B** depicts the hub network constructed using the Cytoscape between the Meliae cortex’s active phytonutrients and the 41 potential anti-COVID-19 core targets (determined in section 3.5). The hub network consisted of 56 nodes and 131 edges. Furthermore, the network diameter, radius, and density were all 7, 4, and 0.067, respectively. The findings of the hub network indicate that all fifteen Meliae cortex’s active phytonutrients interact with 41 potential anti-COVID-19 core targets to a different degree.

**Figure 8:**
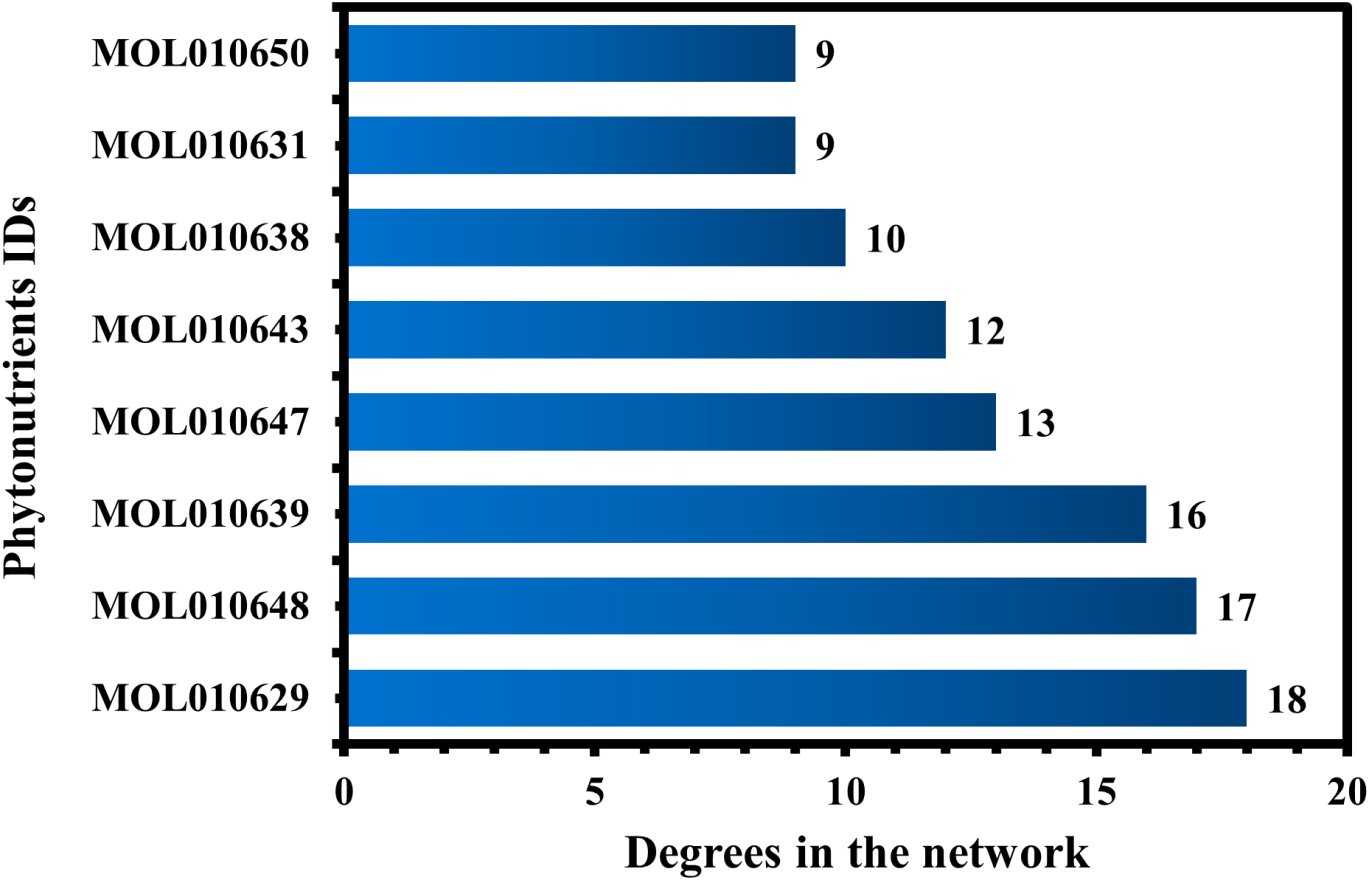
The top eight Meliae cortex’s key active phytonutrients ranked with their degree values in the hub network.

MOL010629 (4,8-dimethoxy-1-vinyl-beta-carboline), MOL010648 (Trichilinin D), MOL010639 (Nimbolin B), MOL010647 (Trichilinin B), MOL010643 (Sendanolactone), MOL010638 (Nimbolin A), MOL010631 (Kulactone), and MOL010650 (SMR000232316) are the top eight active phytonutrients ranked by their DC ≥ average value (8.437) that interact with more than eight potential anti-COVID-19 core targets in the hub network. These are recognized as key active phytonutrients of the Meliae cortex and are exhibited in the form of a bar graph ranked by DC in the hub network (**Figure 8**). Consequently, our findings confirmed the synergistic mode of interaction between the multiple Meliae cortex’s active phytonutrients and the multiple anti-COVID-19 core targets in treating COVID-19.

### 3.8 GO enrichment analysis

**Figure 9:**
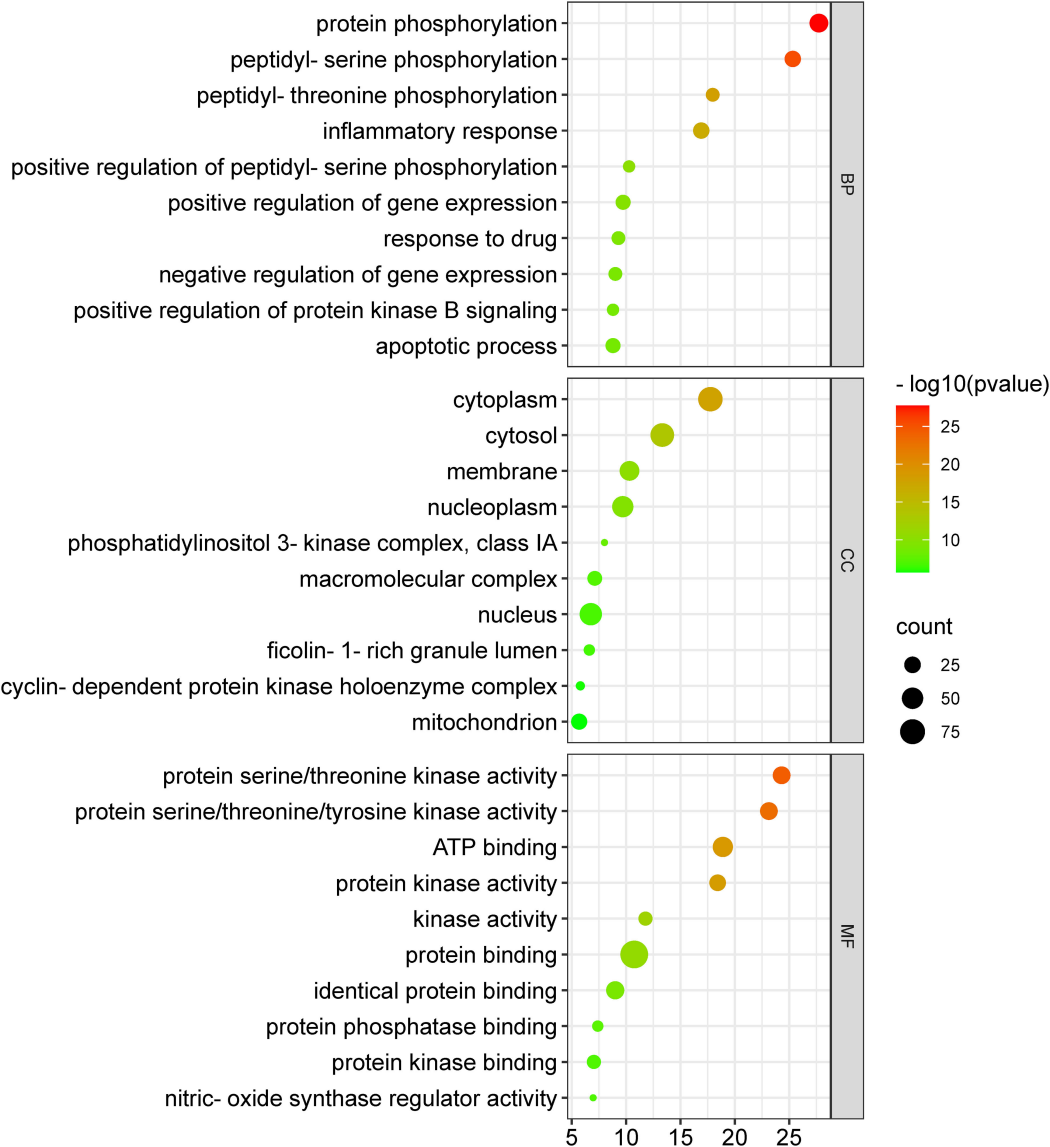
GO enrichment analysis of 104 potential anti-COVID-19 prime targets. The number of gene targets is shown on the X-axis, while the MF, CC, and BP are represented on the Y-axis.

The 104 potential anti-COVID-19 prime targets were further analyzed for GO enrichment analysis. The top 10 enrichment terms of MF, CC, and BP of 104 potential anti-COVID-19 prime targets are shown in **Figure 9**. GO enrichment analysis shows that the gene targets related to BP are involved in protein phosphorylation, peptidyl-serine phosphorylation, inflammatory response, apoptotic process, negative regulation of gene expression, etc. Gene targets in CC are mainly involved in the cytoplasm, cytosol, nucleus, nucleoplasm, membrane, mitochondrion, etc. GO enrichment analysis further demonstrates that the enriched MF ontology is dominated by protein binding, ATP binding, protein serine/threonine kinase activity, protein serine/threonine/tyrosine kinase activity, identical protein binding, protein kinase activity, etc.

### 3.9 KEGG analysis

**Figure 10:**
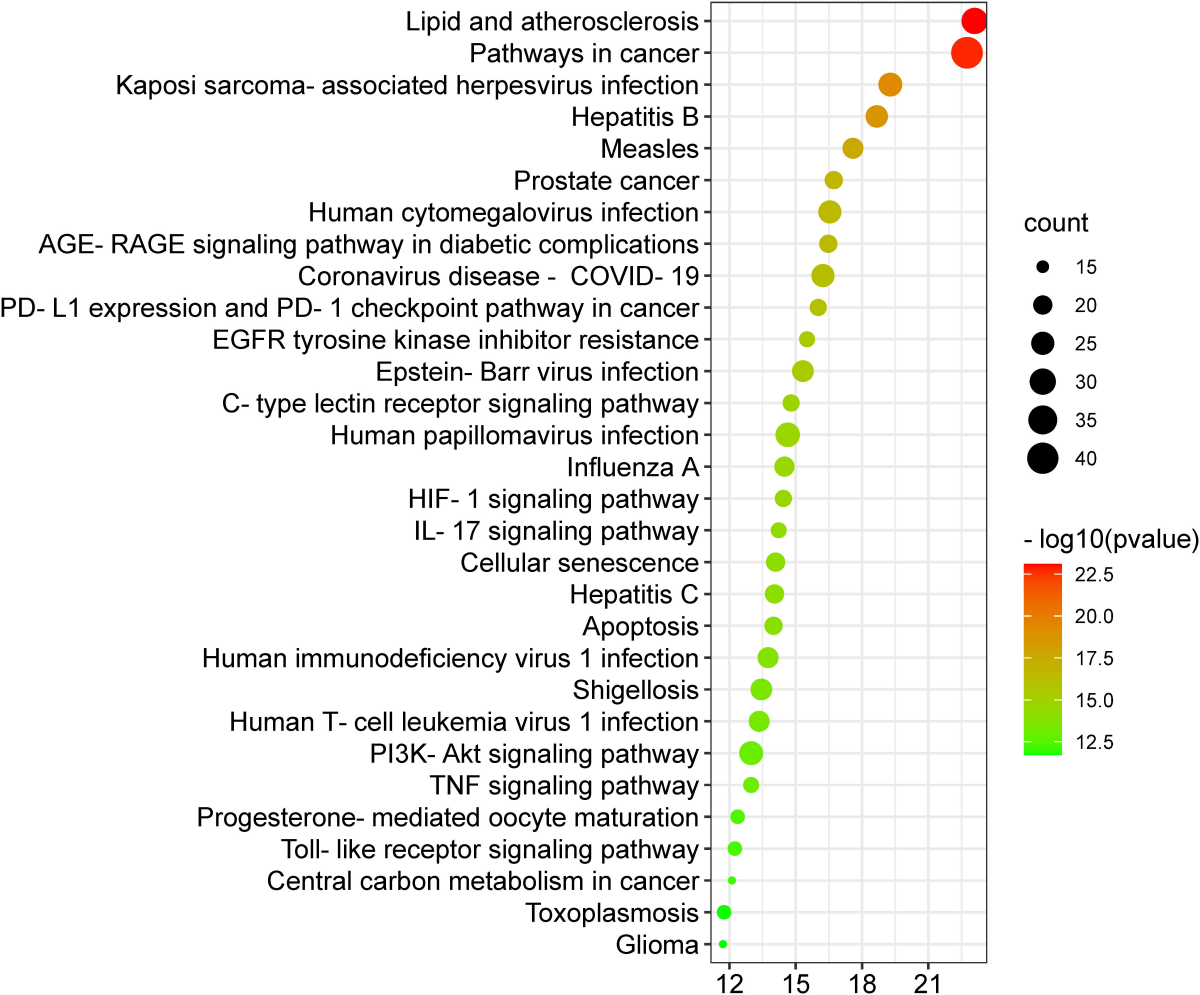
Top 30 KEGG pathways. X-axis denotes the total number of genes, Y-axis displays the multiple KEGG pathways, and the bubble-size shows the gene numbers involved in each KEGG pathway.

A KEGG pathway analysis was executed to ascertain the pharmacological mechanisms through which the Meliae cortex alleviates COVID-19. After uploading 104 potential anti-COVID-19 prime targets to the DAVID platform, 158 pathways with *p* < 0.05 were identified. **Figure 10** shows the top 30 important KEGG pathways. The enrichment analysis revealed that anti-COVID-19 targets of the Meliae cortex might be involved in pathways in cancer, lipid and atherosclerosis, Coronavirus disease – COVID-19, human cytomegalovirus infection, PI3K-Akt signaling pathway, HIF-1 signaling pathway, IL-17 signaling pathway, TNF signaling pathways, EGFR tyrosine kinase inhibitor resistance, etc. These pathways may all make a substantial contribution to the molecular mechanism of the Meliae cortex in treating COVID-19.

### 3.10 Core pathways determination

**Figure 11:**
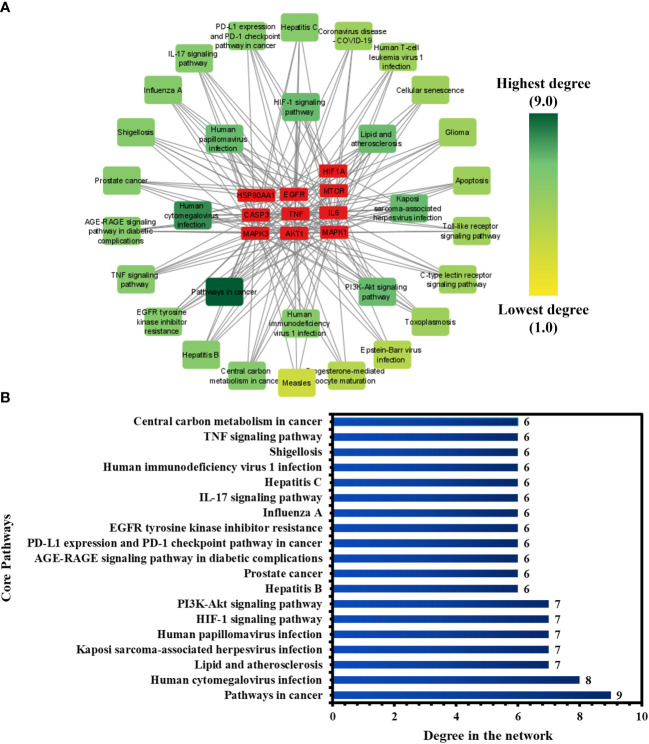
**(A)** Network of pathways and anti-COVID-19 core targets. The hue shifts from yellow to green with an increasing degree of a pathway. **(B)** Nineteen core pathways with their degree values in the network.

To investigate the core pathways contributing to the anti-COVID-19 effects of the Meliae cortex’s key active phytonutrients, a network was developed by integrating the top thirty KEGG pathways and the top ten anti-COVID-19 core targets (**Figure 11A**). The findings demonstrate that the network has 40 nodes and 175 edges. Furthermore, the network diameter, radius, and density were all 4, 3, and 0.213, respectively. The findings of the network indicate that all thirty pathways interact with ten anti-COVID-19 core targets to a different degree. The maximum degree (9.0) was presented by pathways in cancer, while measles displayed the lowest degree (3.0). The color of the pathways’ node changes from yellow to green as the degree increases (**Figure 11A**).

Furthermore, pathways in the network were ranked by DC ≥ average value of (5.833), and nineteen core pathways were identified, as displayed in **Figure 11B**. Nine of ten anti-COVID-19 core targets (HIF1A, EGFR, CASP3, AKT1, MAPK1, MAPK3, HSP90AA1, mTOR, and IL-6) followed the pathways in cancer. On the other hand, eight out of ten anti-COVID-19 core targets (TNF, EGFR, CASP3, AKT1, MAPK1, MAPK3, mTOR, and IL-6) followed the human cytomegalovirus infection. Hence, findings show that these nineteen core pathways potentially contribute to the anti-COVID-19 effects of the Meliae cortex’s key active phytonutrients.

### 3.11 Molecular docking investigations

**Figure 12:**
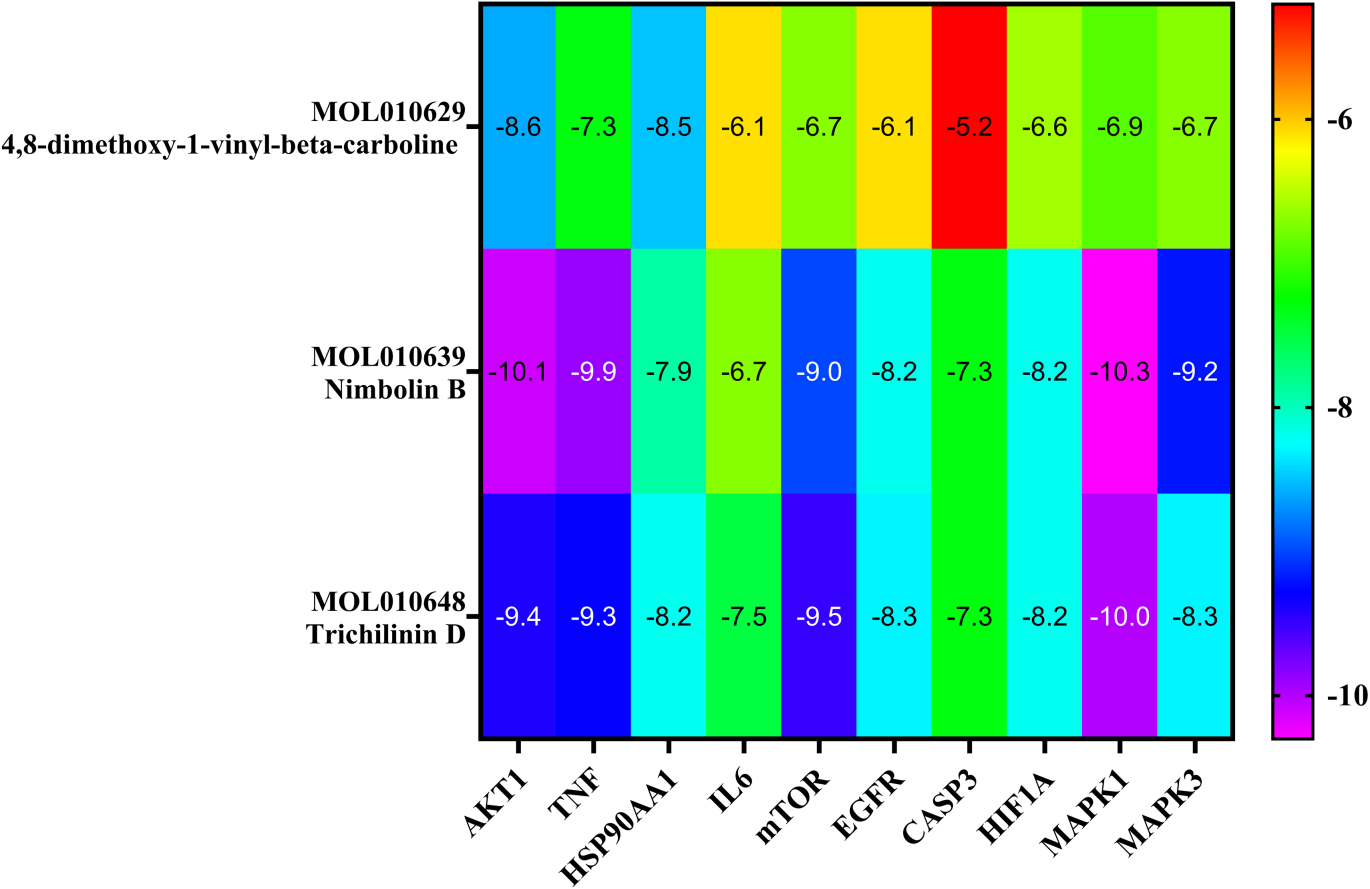
The heatmap exhibits binding affinity (kcal/mol) scores for three of the Meliae cortex’s key active phytonutrients with the top ten anti-COVID-19 core targets. The hue transitions from purple to red as the binding affinity scores decrease.

**Figure 13:**
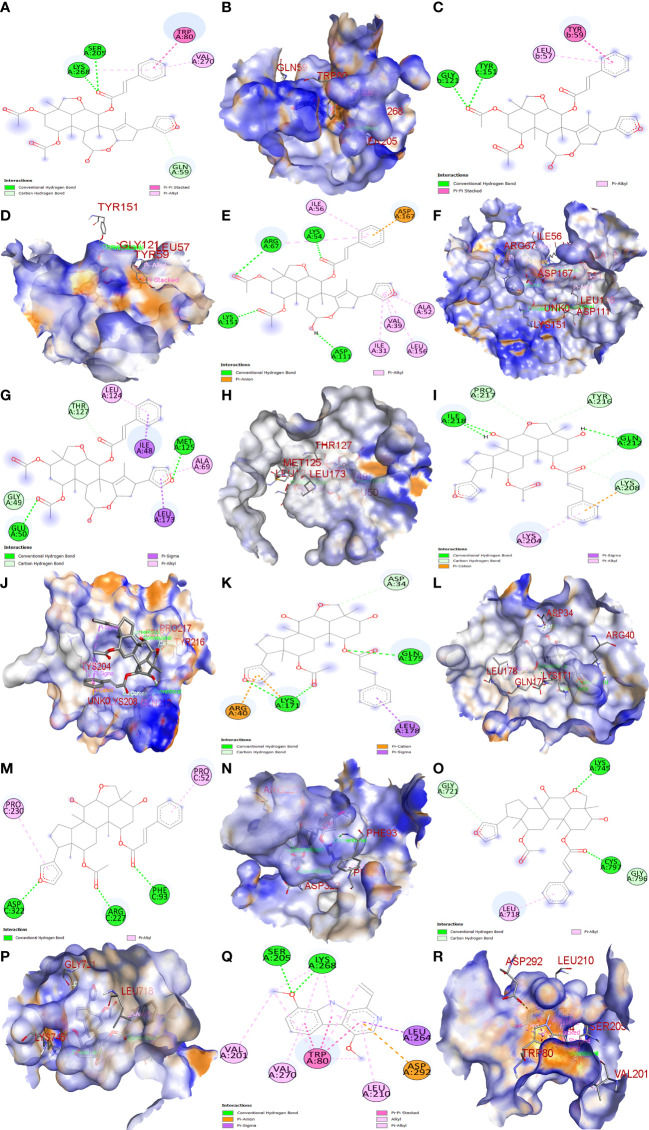
Molecular docking results. Binding of MOL010639 (Nimbolin B) with **(A**, **B)** (2D & 3D) AKT1, **(C**, **D)** (2D & 3D) TNF, **(E**, **F)** (2D & 3D) MAPK1, and **(G**, **H)** (2D & 3D) MAPK3. Binding of MOL010648 (Trichilinin D) with **(I**, **J)** (2D & 3D) HSP90AA1, **(K**, **L)** (2D & 3D) IL-6, **(M**, **N)** (2D & 3D) mTOR, and **(O**, **P)** (2D & 3D) EGFR. Binding of MOL010629 with **(Q**, **R)** (2D & 3D) (4,8-dimethoxy-1-vinyl-beta-carboline).

The top three Meliae cortex’s key active phytonutrients were further analyzed for molecular docking with the top ten anti-COVID-19 core targets, including AKT1, TNF, HSP90AA1, IL-6, mTOR, EGFR, CASP3, HIF1A, MAPK3, and MAPK1. The results of molecular docking analysis in terms of binding affinity (kcal/mol) scores are presented in the form of a heatmap, as shown in **Figure 12**. Furthermore, their docked complexes are shown in 2D and 3D forms in **Figures 13A–R**. The lower binding affinity represents the phytonutrients’ and targets’ superior binding ability (56). The findings showed that all three Meliae cortex’s key active phytonutrients had good to excellent binding affinity scores (< -5.0 to < -7.0 kcal/mol), respectively, with all anti-COVID-19 core targets. However, MOL010639 (Nimbolin B) presented superior binding affinity scores (-10.3, -10.1, -9.9, and -9.2 kcal/mol) with MAPK1, AKT1, TNF, and MAPK3, respectively. On the other hand, MOL010648 (Trichilinin D) demonstrated superior binding affinity scores (-8.2, -7.5, -9.5, and -8.3 kcal/mol) for HSP90AA1, IL-6, mTOR, and EGFR, respectively. In addition, both MOL010639 (Nimbolin B) and MOL010648 (Trichilinin D) exhibited similar binding affinity scores (-7.3 and -8.2) against CASP3, and HIF1A, respectively. Although MOL010629 (4,8-dimethoxy-1-vinyl-beta-carboline) presented inferior binding affinity scores compared to MOL010639 (Nimbolin B) and MOL010648 (Trichilinin D), it has exhibited excellent binding affinity scores < -7.0 kcal/mol against AKT1, TNF, and HSP90AA1. Moreover, MOL010629 (4,8-dimethoxy-1-vinyl-beta-carboline) displayed good binding affinity scores < -5.0 kcal/mol against IL-6, mTOR, EGFR, CASP3, HIF1A, MAPK1, and MAPK3. Hence, the findings of molecular docking analysis corroborated that the Meliae cortex may ameliorate COVID-19 disease by modulating the functions/expressions of these targets.

## 4 Discussion

The current research work investigated the key active phytonutrients, molecular targets, and molecular mechanisms of the Meliae cortex in alleviating COVID-19 disease. In all, fifteen phytonutrients were retrieved based on OB ≥ 30% and DL ≥ 0.10 and were considered active phytonutrients in the Meliae cortex, as given in **Table 1**. MOL010629 (4,8-dimethoxy-1-vinyl-beta-carboline), MOL010648 (Trichilinin D), MOL010639 (Nimbolin B), MOL010647 (Trichilinin B), MOL010643 (Sendanolactone), MOL010638 (Nimbolin A), MOL010631 (Kulactone), and MOL010650 (SMR000232316) are the top eight active phytonutrients ranked by their DC ≥ average value (8.437) that interact with more than eight potential anti-COVID-19 core targets in the hub network. They are recognized as key active phytonutrients. The network findings also revealed a synergistic/combined effect of multiple anti-COVID-19 core targets and multiple key active phytonutrients in the Meliae cortex in alleviating COVID-19 pathology (6, 57).

**Figure 14:**
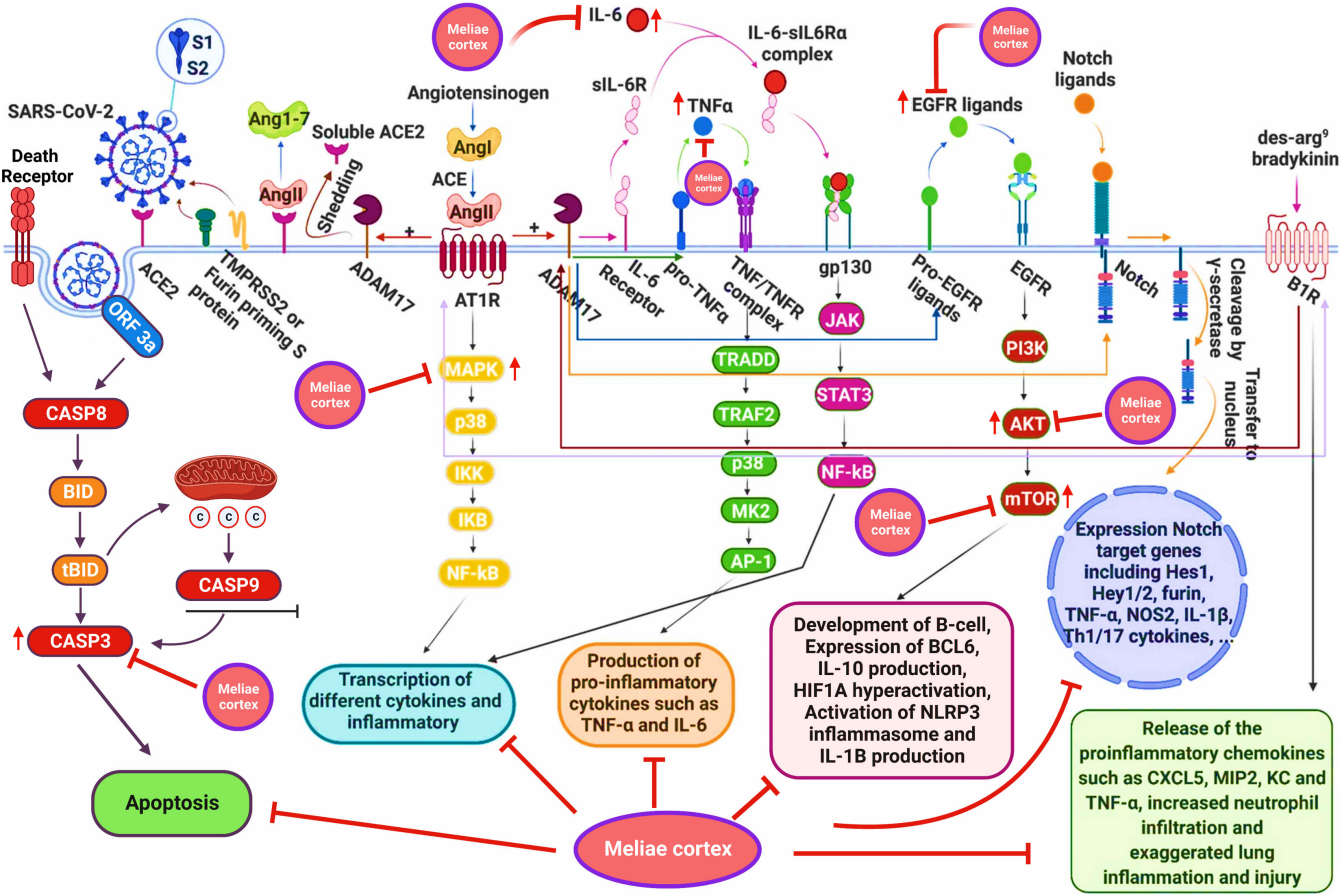
Schematic presentation for the molecular mechanism of COVID-19 and possible anti-COVID-19 mechanisms of the Meliae cortex. Meliae cortexpotentially inhibit multiple molecular targets (IL-6, TNF-a, MAPK3, MAPK1, AKT1, mTOR, HIF1A, EGFR, CASP3, etc.) and molecular pathways (PI3K-Aktsignaling pathway, HIF-1 signaling pathway, etc.) for combating COVID-19. Figure was adapted from (61) and created with BioRender..

The PPI network analysis exhibits that numerous genes, including IL-6, TNF-α, MAPK3, MAPK1, AKT1, mTOR, HIF1A, HSP90AA1, EGFR, CASP3, etc., are implicated in the pathogenicity of COVID-19 disease and also in the anti-COVID-19 effects of Meliae cortex’s key active phytonutrients (**Figures 4B**, **5**, and **14**). Recent reports demonstrate that IL-6 and TNF-α were overexpressed in COVID-19 patients, causing inflammation. TNF-α and IL-6-mediated CRS causes significant acute respiratory distress in COVID-19 patients (6, 12). MAPK1 and MAPK3 overactivation promote CRS by releasing pro-inflammatory cytokines such as IL-1, IL-6, IL-10, TNF-α, IL-4, and IFN-γ. In COVID-19 patients, their hyperactivation also results in vascular endothelial infestations, thrombotic events, alveolar tissue damage, and acute lung injury (22, 58–60). Targeting TNF-α, IL-6, MAPK1, and MAPK3 might be potential alternative treatments for CRS and COVID-19 (**Figure 14**). Dose-dependent activation of AKT1 has reportedly been seen during SARS-CoV-2 infection (62, 63). mTOR hyperactivation during SARS-CoV-2 infections led to immunopathology by promoting Th17 differentiation and inhibiting Treg development. COVID-19 pathology may be averted by AKT1 and mTOR inhibitors (64, 65). Tian et al. demonstrated that the overactivation of HIF1A by the viral ORF3a protein during SARS-CoV-2 infection caused a significant inflammatory response (66). Mills et al. also reported that when macrophages are activated, metabolic changes shift mitochondria from ATP production to ROS formation, resulting in a pro-inflammatory state through the upregulation of HIF1A (67). HSP90AA1 plays a vital role in viral replication. A study reported by Wyler et al. indicated that inhibiting HSP90AA1 not only suppresses SARS-CoV-2 replication but also dampens the induction of inflammatory cytokines such as CXCL11, CXCL10, and IL-6 (68). Numerous lung cells reportedly express EGFR after exposure to SARS-CoV-2 infections, and its amplification aggravates pulmonary illnesses and induces fibrosis (69). Compared to healthy individuals, CASP3 was shown to be upregulated in the red blood cells of COVID-19 patients. In addition, CASP3 is involved in the programmed cell death of platelets and white blood cells, which leads to thrombotic events and leukopenia, respectively, in COVID-19 patients with chronic illness (61, 70, 71). Thus, suppressing EGFR and CASP3 may alleviate pulmonary disease and fibrosis, as well as thrombotic events and leukopenia, respectively, which play a significant part in the pathophysiology of COVID-19 (**Figure 14**).

GO enrichment analysis shows that anti-COVID-19 effects of Meliae cortex’s key active phytonutrients might be attributed to multiple gene targets that are associated with multiple BP such as protein phosphorylation, peptidyl-serine phosphorylation, inflammatory response, apoptotic process, positive regulation of gene expression, negative regulation of gene expression, positive regulation of protein kinase B signaling, etc. Reports demonstrate that the SARS-CoV-2 infection stimulates host kinases, causing high phosphorylation in the host and virus. Thus, targeting host human kinases may result in innovative therapies (72). Many studies indicate that after the invasion of SARS-CoV-2, the hyperactivation of the innate and adaptive immune systems induces an inflammatory response. This further stimulates several intracellular cytokines, such as NF-κB, mTOR, TNF- α, etc., that contribute to COVID-19’s pathophysiology (6, 12). Apoptosis is a process of programmed cell death that the host may induce to limit virus replication. Excessive apoptosis damages the bronchoalveolar network, causing lung injury and ARDS. It is known that coronaviruses trigger apoptosis in various ways. SARS-CoV-2 ORF3a activated CASP8/CASP9/BID, triggering apoptosis (**Figure 14**). SARS-CoV ORF6 and SARS-CoV-2 ORF6 promote apoptosis through CASP3 and endoplasmic reticulum stress (73, 74). The anti-COVID-19 effects of the Meliae cortex’s key active phytonutrients might be attributable to a variety of gene targets found mostly in the cytoplasm, cytosol, nucleus, nucleoplasm, membrane, mitochondrion, etc. Enriched MF ontology analysis reveals that these multiple gene targets execute various functions such as protein binding, ATP binding, protein serine/threonine kinase activity, protein serine/threonine/tyrosine kinase activity, identical protein binding, protein kinase activity, etc.

The KEGG pathway enrichment analysis exhibited that the anti-COVID-19 effects of the Meliae cortex’s key active phytonutrients could be associated with pathways in cancer, lipid and atherosclerosis, Coronavirus disease – COVID-19, human cytomegalovirus infection, PI3K-Akt signaling pathway, HIF-1 signaling pathway, IL-17 signaling pathway, TNF signaling pathways, EGFR tyrosine kinase inhibitor resistance, etc. Moreover, the network analysis of the top thirty KEGG pathways and top ten anti-COVID-19 core targets identified the nineteen core pathways ranked by DC ≥ average value of (5.833), as shown in **Figure 11B**. Among them, pathways in cancer, human cytomegalovirus infection, HIF-1 signaling pathway, and PI3K-Akt signaling pathway are the core pathways followed by nine of ten anti-COVID-19 core targets (HIF1A, EGFR, CASP3, AKT1, MAPK1, MAPK3, HSP90AA1, mTOR, and IL-6), eight out of ten anti-COVID-19 core targets (TNF, EGFR, CASP3, AKT1, MAPK1, MAPK3, mTOR, and IL-6), 7 of ten anti-COVID-19 core targets (HIF1A, EGFR, mTOR, IL-6, AKT1, MAPK1, and MAPK3), and 7 of ten anti-COVID-19 core targets (EGFR, AKT1, MAPK1, MAPK3, HSP90AA1, mTOR, and IL-6), respectively. Results show that pathways in cancer regulation may contribute to the modulation of anti-COVID-19 targets. Targeting cancer-related pathways may also affect their expression in COVID-19. Hayashi et al. showed that anticancer medicines block MAPK and suppress SARS-CoV-2 proliferation (75). Numerous studies have demonstrated the contribution of the HIF-1 signaling pathway to SARS-CoV-2 infection, which exacerbates COVID-19-induced inflammatory responses and CRS (66). Moreover, hyperactivation of the PI3K-Akt signaling pathway is involved in viral entry into cells and in the induction of pro-inflammatory mediators (TNF-α, IL-6, etc.) that significantly contribute to the etiology of COVID-19 (**Figure 14**) (76). Hence, findings of current investigations show that these core pathways potentially contribute to the anti-COVID-19 effects of the Meliae cortex’s key active phytonutrients.

## 5 Conclusion

In this work, we effectively explored the molecular targets, molecular pathways, and key active phytonutrients of the Meliae cortex for the treatment of COVID-19. The 104 potential anti-COVID-19 prime targets, 41 potential anti-COVID-19 core targets, and eight key active phytonutrients were determined. The top 10 of 41 potential anti-COVID-19 core targets (AKT1, TNF, HSP90AA1, IL-6, mTOR, EGFR, CASP3, HIF1A, MAPK3, and MAPK1) were identified as molecular targets of key active phytonutrients of the Meliae cortex that might be implicated in ameliorating COVID-19. We have further identified that the mechanism of action of the Meliae cortex’s key active phytonutrients for anti-COVID-19 therapy might be the suppression or modulation of several biological processes, such as phosphorylation, inflammatory response, and apoptotic process. Furthermore, three molecular pathways out of 19 core pathways were identified, such as the PI3K-Akt signaling pathway, the HIF-1 signaling pathway, and the pathways in cancer, that significantly contribute to modulating molecular targets and thereby alleviating COVID-19 with Meliae cortex’s key active phytonutrients. The findings of molecular docking analysis further corroborated that the Meliae cortex may ameliorate COVID-19 disease by modulating the functions/expressions of these targets. Consistency in results was found between the findings of network pharmacology and molecular docking, thereby corroborating the validity of network pharmacology. Hence, this research offers a solid theoretic foundation for the future developments of anti-COVID-19 therapeutics based on the phytonutrients of the Meliae cortex.

## Data availability statement

The raw data supporting the conclusions of this article will be made available by the authors, without undue reservation.

## Author contributions

SK and TL: validation, investigation, and writing—review and editing and original draft preparation. SK: conceptualization, methodology, software, formal analysis, data curation, and visualization. TL: supervision, project administration, and funding acquisition. All authors contributed to the article and approved the submitted version.
